# Higher Levels of Psychological Burden and Alterations in Personality Functioning in Crohn’s Disease and Ulcerative Colitis

**DOI:** 10.3389/fpsyg.2021.671493

**Published:** 2021-06-24

**Authors:** Felicitas Engel, Sabrina Berens, Annika Gauss, Rainer Schaefert, Wolfgang Eich, Jonas Tesarz

**Affiliations:** ^1^Department of General Internal Medicine and Psychosomatics, University Hospital Heidelberg, Heidelberg, Germany; ^2^Institute of Psychology, Heidelberg University, Heidelberg, Germany; ^3^Department of Gastroenterology and Hepatology, University Hospital Heidelberg, Heidelberg, Germany; ^4^Division of Internal Medicine, Department of Psychosomatic Medicine, University Hospital Basel, Basel, Switzerland

**Keywords:** crohn’s disease, ulcerative colitis, operationalized psychodynamic diagnostics, psychodynamic structural characteristics, personality functioning levels, psychological burden

## Abstract

**Aims**: Is there evidence for increased psychological distress and alterations in personality functioning in patients with Crohn’s disease (CD) and ulcerative colitis (UC) compared to healthy controls (HCs)?

**Background**: In patients with CD and UC, perceived stress is closely associated with changes in disease activity. The stress response is influenced by psychological burden and personality functioning, but only little is known about these factors in inflammatory bowel diseases (IBD).

**Study**: A total of 62 patients with an endoscopic ensured CD/UC without remission (*n* = 31 per group) and 31 HC were included. Patients with an active CD/UC and HC were individually matched (*n* = 93, 31 per group) for age, sex, education, and disease activity. Depression and anxiety were assessed to evaluate the effect of psychological burden (Patient Health Questionnaire-9/PHQ-9, Generalized Anxiety Disorder-7/GAD-7). Personality functioning was measured by validated questionnaires for psychodynamic structural characteristics, mentalization, and attachment (Operationalized Psychodynamic Diagnosis-Structure Questionnaire/OPD-SQ, Mentalization Questionnaire/MZQ, and Experiences in Close Relationships scale/ECR-RD 12).

**Results**: Levels of depression and anxiety were higher in CD/UC patients than in HC with large effect sizes. Comparing personality functioning in CD/UC with HC, psychodynamic structural characteristics differed between CD/UC and HC with medium effect sizes, with structural differences occurring primarily in the domain of self-perception and regulation. Only minor differences were found regarding mentalization and attachment. CD and UC differed only with small effect sizes.

**Conclusion**: Our data show that compared to HC, patients with CD/UC are characterized by a higher level of psychological burden and structural alterations in the domain of self.

## Introduction

Inflammatory bowel diseases (IBD) affect 2.5–3 million people in Europe with direct healthcare costs of 4.6–5.6 billion Euros/year (Burisch et al., 2013). The most common IBD are Crohn’s disease (CD) and ulcerative colitis (UC), and the incidence and prevalence of these illnesses are increasing in Europe (Burisch et al., 2013). Despite the high prevalence of these conditions, the pathogenesis of IBD is still not well understood but is widely thought to be multifactorial (de Souza, 2017).

While research into biological and microbiotic factors is currently a strongly growing field of research, the investigation of personality factors is an area that is not new, but still controversial. Over 70% of the IBD patients, however, believe that stress or their own personality are relevant contributors within a biopsychosocial disease model of IBD (Sajadinejad et al., 2012; Bernstein, 2017). There is reasonable suspicion for a biomechanistic link between stress and IBD: psychosocial stress results in the activation of the enteric nervous system, which may lead to an increase in levels of proinflammatory cytokines with a subsequent inflammatory relapse resulting in symptom exacerbation in IBD (Singh et al., 2009). With regard to the psychological burden of anxiety and depression, the direction of (neuro-)inflammatory processes and disease activity in CD/UC seems to be bidirectional – with patients with anxiety symptoms developing more often disease flares, and patients with disease activity developing more anxiety (Gracie et al., 2018). Additionally, recent neuroimaging studies have shown that patients with (active) IBD have an altered brain structure, which is associated with symptoms of depression and anxiety (Agostini et al., 2011, 2013a,b, 2017; Vogt, 2013; Zikou et al., 2014; Kragel et al., 2018; Hou et al., 2019; Colonnello and Agostini, 2020; Fan et al., 2020; Skrobisz et al., 2020; Thomann et al., 2020; Turkiewicz et al., 2021; Yeung, 2021).

Interestingly, the association between the activity of IBD and stress seems to be particularly for perceived stress, less for the simple presence of stressful life events (Singh et al., 2009). Perceived stress is associated with an increased risk of exacerbation in UC with objective (mucosal abnormalities in endoscopy) and subjective (e.g., increased frequency of bowel movements) disease markers (Levenstein et al., 2000). Key features that influence our response to stressors and our individual coping with stress are determined by the characteristics of our personality functioning.

Personality functioning describes how an individual typically experiences himself or herself as well as others and includes usually dimensions of self-functioning and interpersonal functioning (Muskin, 2014). First evidence of differences in personality functioning in individuals with high levels of emotional distress has been demonstrated (Ehrenthal et al., 2012; Ladwig, 2016; Schojai, 2017; Tasca et al., 2018; Fischer-Kern and Tmej, 2019), and the question is whether these differences can also be found in patients with medical diseases that may be associated with increased perceived stress, like IBD. Important concepts related to stress response and coping with life stressors focus on psychodynamic structural characteristics, mentalization skills, and attachment style (Mikulincer and Shaver, 2003; Zimmermann et al., 2012; Hayden et al., 2018).

Psychodynamic structural characteristics describe the person’s internal and interpersonal abilities to shape their own life in a proper manner and to adapt to change. Required skills are perception/cognition, regulation, communication, and attachment – all inside the self and outside (Zimmermann et al., 2012). For example, for the self and internal world, self-perception, self-regulation, internal communication, and attachment to internal objects play an important role. Higher levels of structural integration seem to be associated with lower self-reported general distress and interpersonal problems (Zimmermann et al., 2012). The relationship between (di-)stress and somatic and mental illnesses has long been known (Kielholz, 1977; Szabo et al., 2017). New assessment instruments allow to thoroughly explore also potentially involved psychodynamic factors (Ehrenthal et al., 2012; Berens et al., 2019).

In addition to psychodynamic structural characteristics, mentalization skills and attachment are the important constructs related to personality functioning. Mentalization is the ability to understand one’s own behavior and the behavior of others with regard to underlying mental states, e.g., feelings, needs, or reasons (Fonagy and Target, 2006). This ability is discussed to play an important role when coping with stress and preventing depressive symptoms (Luyten and Fonagy, 2018). Chronic stress – which is what chronic exposure to physical discomfort in the context of IBD usually represents – may lead to an inhibition of mentalization processes. This in turn might result in difficulties in interpersonal relationships and the development of psychological comorbidities, as well as problems in patient–physician interactions and decreased medication adherence (Agostini et al., 2019; Colonnello and Agostini, 2020). This hypothesis is further supported by neuroimaging studies of patients with IBD, which found alterations in brain areas (e.g., the midcingulate cortex), that are involved in emotion processing (Agostini et al., 2011, 2013a,b, 2017; Vogt, 2013; Zikou et al., 2014; Kragel et al., 2018; Hou et al., 2019; Colonnello and Agostini, 2020; Fan et al., 2020; Skrobisz et al., 2020; Thomann et al., 2020; Turkiewicz et al., 2021; Yeung, 2021). This is in line with previous studies that reported that IBD patients are characterized by mentalizing deficits compared to healthy controls (HCs). However, these studies did not distinguish between CD and UC (Agostini et al., 2019; Berens et al., 2019). Furthermore, chronic stress, like in IBD, is believed to affect patient’s mentalizing abilities and to determine a shift toward attachment insecurity in patients (Agostini et al., 2019).

Attachment describes the type of bond that an individual has with others; this is usually due to the type of bond to early reference persons, especially the parents (Caplan et al., 2014). The attachment style is another important influencing factor with regard to interpersonal functioning and stress. For example, individuals with an anxious attachment style find various stressors more threatening, which can intensify the experience of stress (Mikulincer and Shaver, 2003). Previous studies showed that the attachment style can be relevant in IBD, while the expression may differ in CD and UC. Specifically, an avoidant attachment style in UC is a moderator between negative childhood experiences and disease activity, while this association could not be shown in CD (Caplan et al., 2014). UC patients seem to have more attachment-related anxiety than HC and therefore experience more perceived stress, while another study found especially CD to be associated with higher levels of emotional distress than UC or HC (Agostini et al., 2016; Petruo et al., 2019). Deficits in mentalization and attachment insecurity may lead, *via* a decreased medical adherence and a decreased social support, to a worsening of the IBD course and afterward to more IBD-related stress and further deficits in a vicious circle (Colonnello and Agostini, 2020). The previous studies showed that stress, personality functioning, or emotional distress can be relevant in IBD, while the expression may differ in UC and CD, and their relevance is still unclear.

Overall, the literature on personality functioning in IBD is limited. The research to date has tended to focus on attachment, mentalization, and the psychological burden of anxiety and depression, rather than exploring the role of the different facets of personality functioning more deeply. Moreover, the studies tend to address only UC/CD against HC, instead of comparing the two IBD with each other.

To overcome some of these shortcomings, this exploratory study describes for the first time psychological burden and personality functioning in patients with CD, UC, and HC using a comprehensive and validated psychological assessment and an objective evaluation of clinical IBD diagnosis. Based on the current data, we postulated that IBD patients are characterized by a higher psychological burden than HC. Regarding the psychodynamic structural characteristics, we did not have *a priori* hypothesis, which is why we chose a primarily exploratory approach.

## Materials and Methods

The study was part of a large multicenter case–control study investigating psychodynamic characteristics of persistent (gastrointestinal) bodily complaints (Berens et al., 2019). The project was approved by the ethics committee of the University of Heidelberg and registered by the German Clinical Trials Register (DRKS; DRKS00011685). Funding was obtained by the Köhler Foundation. Data are available on reasonable request.

### Patient Recruitment and Inclusion/Exclusion Criteria

The study included 31 individually matched patients with CD and UC, respectively (62 IBD patients in total) and 31 individually matched HC (matching criteria: age, gender, educational level, and disease activity, see also statistical analysis). In order to define a patient sample that is as homogeneous and valid as possible, we decided *a priori* to apply very strict inclusion criteria at the expense of the sample size. IBD patients were recruited between February and December 2017 in primary care (general practitioners’ practices), secondary care (gastroenterological specialty practices), and tertiary care (University Hospital) in Heidelberg/Germany (Berens et al., 2019). Inclusion criteria for CD/UC were as follows: the presence of an IBD [CD or UC, no indeterminate (unclassified) colitis], confirmation of IBD diagnosis by a physician (previous endoscopic examination was mandatory), and active state of disease [according to Manitoba IBD Index (MIBDI); Clara et al., 2009]. To ensure a broad recruitment strategy in primary, secondary, and tertiary care of patients with gastrointestinal symptoms and to improve patient compliance, no further diagnostics were performed (e.g., collection of tissue samples, blood, or stool tests). HC were recruited *via* an online opportunity sampling by SociSurvey (Leiner, 2014). HC were screened *via* a questionnaire for somatic comorbidities (Charlson comorbidity index; Charlson et al., 1987) and medication intake, and excluded from the “HC” sample, if they indicated any somatic or psychiatric disease or if they used any antidepressants.

### Measurements

The study participants completed a questionnaire set containing sociodemographic, physical, and psychological characteristics.

#### Sociodemographic Characteristics

The psychosomatic basis documentation questionnaire (Psy-BaDo) was used to assess information about age, gender (female/male), nationality (German/other), marital status (living with a partner; yes/no), educational level (International Standard Classification of Education, ISCED ≤ 2), and professional life (paid employment/disability pension/old-age pension; Heuft et al., 1998).

#### Clinical Symptom Severity

The MIBDI was used to measure the level of symptom severity (Clara et al., 2009). The MIBDI assesses the symptom severity of IBD for the previous 6 months from constantly active to not active at all and is validated for CD and UC (Clara et al., 2009). For better comparability, IBD patients were excluded if they did not meet the criteria for active disease (constantly active disease with symptoms every day to occasionally active with symptoms 1–2 days/month).

#### Psychological Burden

Psychological burden was measured by assessing the levels of depressive and anxiety-related complaints.

##### Depressive Complaints

Depressive complaints were examined with the depression module of the Patient Health Questionnaire (PHQ-9; Kroenke et al., 2001). The questionnaire uses nine items for depression on a four-point scale (0–3). A depressive complaints score can be calculated from 0 to 27. Overall, 0–4 is for minimal, 5–9 for mild, 10–14 for moderate, 15–19 for moderately severe, and 20–27 for severe depressive symptoms (Kroenke et al., 2001). Besides the score for depressive symptoms, the presence of a major depressive syndrome can be calculated (Löwe et al., 2002). The questionnaire is validated for phenotyping patients with gastrointestinal diseases (Persoons et al., 2001) and showed good reliability (Cronbach’s *α* = 0.835) in our study.

##### Anxiety

Anxiety was measured using the Generalized Anxiety Disorder Questionnaire (GAD-7). The sum score of seven items (0–21) can be divided into minimal (0–4), mild (5–9), moderate (10–14), and severe (15–21) level of anxiety severity (Spitzer et al., 2006). In addition, the presence of a generalized anxiety disorder can be calculated (Löwe et al., 2002). The questionnaire is validated for phenotyping patients with gastrointestinal diseases (Persoons et al., 2001) and showed an excellent reliability (Cronbach’s *α* =0.910) in our study.

#### Personality Functioning

Personality functioning was measured by validated questionnaires for psychodynamic structural characteristics, mentalization skills, and attachment.

##### Psychodynamic Structural Characteristics

Psychodynamic structural characteristics were assessed using the operationalized psychodynamic diagnosis (OPD)-Structure Questionnaire (OPD-SQ; Ehrenthal et al., 2012). The OPD-SQ is a questionnaire for the structural axis in the OPD system. It consists of eight primary dimensions. Four refer to the self/internal world – namely, self-perception, self-regulation, internal communication, and attachment to internal objects – and the remaining four relate to objects/external world, namely, object perception, regulation of relationships, external communication, and attachment to external objects.

The primary dimensions can be described as follows: self-perception is the ability to perceive and reflect on oneself: An item is, for example, “I prefer not to think about myself, because all I would face is chaos.” For object-perception, kind of the same applies with regard to the relation to other individuals (inter alia, individuals are referred to as “objects” in psychodynamic theory): an item on this scale is, for example, “I tend to relate others’ remarks or actions to myself which may not really be connected to me at all.” Self-regulation, and regulation of relationships, is the ability to control oneself, for example, in relation to one’s own emotions, toward oneself and others: An item is, for example, “Sometimes my feelings are so intense that I get scared” (self-regulation) or “I frequently cause harm in relationships when I’m angry” (regulation of relationships). Internal communication and external communication describe the contact to oneself and other individuals: Items are, for example, “I often cannot feel my body properly” (internal communication) or “I feel uneasy if I have to approach a stranger” (external communication). Attachment to internal or external objects describes the ability to deal with oneself and others, for example, in relation to intimacy, asking for help, or leaving a relationship. Items are, for example, “I do not treat myself particularly well” (internal) or “I find it difficult to ask others for help” (external; Ehrenthal et al., 2012; Zimmermann et al., 2012).

The OPD-SQ-sum score and the dimensions are calculated as mean values (0–4); higher scores reflect higher deficits in structure (Ehrenthal et al., 2012). The OPD-SQ is a valid instrument for assessing psychodynamic structural characteristics and showed an excellent reliability (Cronbach’s *α* = 0.968) in our study.

##### Mentalization Abilities

Mentalization abilities were assessed by the *Mentalization Questionnaire (MZQ)*. This questionnaire measures mentalizing in mean values in 15 items from 0 (not at all) to 4 (fully agree) with asking, e.g., “Sometimes I only become aware of my feelings in retrospect.” Higher scores indicate a higher deficit in mentalizing (Hausberg et al., 2012). The MZQ showed good reliability (Cronbach’s *α* = 0.850) in our study.

##### Attachment

Attachment was assessed using the short form of the Experiences in Close Relationships Scale (ECR-RD 12; Lafontaine et al., 2016). The questionnaire consists of two dimensions of insecure attachment: attachment-related anxiety (e.g., “I need a lot of reassurance that I am loved by my partner”) and attachment-related avoidance (e.g., “I do not feel comfortable opening up to romantic partners”). In each attachment style, mean values were calculated from six items on a scale of 0 (not at all) to 7 (fully agree). Higher scores reflect higher attachment-related anxiety or avoidance (Lafontaine et al., 2016). The two different scales of the questionnaire (anxiety/avoidance) showed acceptable/good internal reliability (Cronbach’s *α* = 0.763/0.807) in our study.

### Statistical Analysis

The sample was individually matched for age (±7 years), gender (f/m), and educational level (ISCED level ≤/> 2; Unesco Institute for Statistics, 2012). Disease activity differed between CD and UC after the first matching; therefore, we matched additionally in a second step for disease activity (±2) on the MIBDI (Clara et al., 2009). We followed a primarily descriptive approach and focused our interpretation on effect sizes. Values of *p* were only indicated for reasons of completeness, but due to the explorative approach, we did not correct for multiple testing. Metric variables were reported as total values or means with SD if they were normally distributed; otherwise, they were reported as median with interquartile range (IQR). Due to normality violations, group comparisons were made using nonparametric Kruskal–Wallis and Mann–Whitney U-tests. Frequencies were compared with chi^2^-tests. *Post hoc* tests were performed when differences were found in the first analysis. Correlation analyses were performed with Spearman’s rho (_Sp_*r*). Missing values were replaced using the mean value imputation, if their frequency was below 20%. Effect sizes (Cohen’s *d*) were computed (no effect: 0–0.1; small effect: 0.2–0.4; medium effect: 0.5–0.7; large effect: ≥0.8; Cohen, 1998). The sample size (*n* = 93 in three groups) enables to find differences with an effect size of *d* ≥ 0.66 (G*Power calculation with alpha = 0.05 and power = 0.8). All data were analyzed using SPSS 24 for Windows (Ibm Corp., 2016). Effect sizes were calculated online (Lenhard and Lenhard, 2016).

## Results

### Recruitment and Matching

A multicenter case–control study screened 1,110 patients in primary, secondary, and tertiary care for IBD and irritable bowel syndrome (IBS; Berens et al., 2019). Since many patients in the general practitioners’ practices, in particular, consulted the physician because of other complaints (e.g., vaccination, flu), the overall proportion of IBD patients in the total collective was correspondingly small. Of the 1,110 patients, 207 patients fulfilled the criteria for an IBD and were eligible, and 193 had an endoscopically secured IBD and completed the questionnaire set (Berens et al., 2019). Of these 193 patients, 127 met the inclusion criteria of the study [active disease, no indeterminate colitis (unclassified IBD)], 85 for CD, and 42 for UC. All IBD patients came from secondary or tertiary care (CD: 14 patients from secondary care, 17 patients from tertiary care; UC: 17 patients from secondary care, 14 from tertiary care). These patients and the HC (*n* = 249 with no somatic or psychiatric disease) were individually matched for gender, age, educational level, and disease activity. Overall, 31 participants per group remained after matching; for sociodemographic and clinical characteristics, see Table 1.

**Table 1:** Sociodemographic and clinical characteristics.

	CD *n* = 31		UC *n* = 31		HC *n* = 31	
**Sociodemographic characteristics**						
Gender – female^a^	31	16 (51.6)	31	16 (51.6)	31	16 (51.6)
Age – years^b^	31	39.2 (14.7)	31	38.5 (14.0)	31	38.5 (13.3)
Lower educational level (ISCED ≤ secondary)^a^	31	16 (51.6)	31	16 (51.6)	31	16 (51.6)
Nationality – German^a^	30	29 (96.7)	31	31 (100.0)	31	30 (96.8)
Marital status – living with a partner^a^	31	19 (61.3)	31	19 (61.3)	31	16 (51.6)
Professional life, paid employment^a^	30	26 (86.7)	30	27 (90.0)	30	25 (83.3)
**Clinical characteristics**						
IBD disease activity (MIBDI, 1–5)^c^	31	3.0 (1.00)	31	2.0 (2.0)	Not applicable	
Flares, year^c^	18	2.5 (3.0)	21	2.0 (3.0)	Not applicable	
Duration of symptoms (in years)^c^	31	13.0 (14.0)	29	7.0 (10.5)	Not applicable	
Therapy with biologicals^a^	31	17 (54.8)	31	17 (54.8)	Not applicable	

CD, Crohn’s disease; UC, ulcerative colitis; HC, healthy controls; ISCED, International Standard Classification of Education; MIBDI, manitoba-inflammatory bowel diseases-index; *p* value calculated by chi-squared method for frequencies and Kruskal-Wallis-Test for categorical variables, for comparison of two groups M-W-U-Tests were performed.

a absolute numbers and percentages *N* (%).

b mean values and standard deviations *M* (*SD*).

c median and interquartile range (IQR).

### Psychological Burden and Personality Functioning

**Figure 1:**
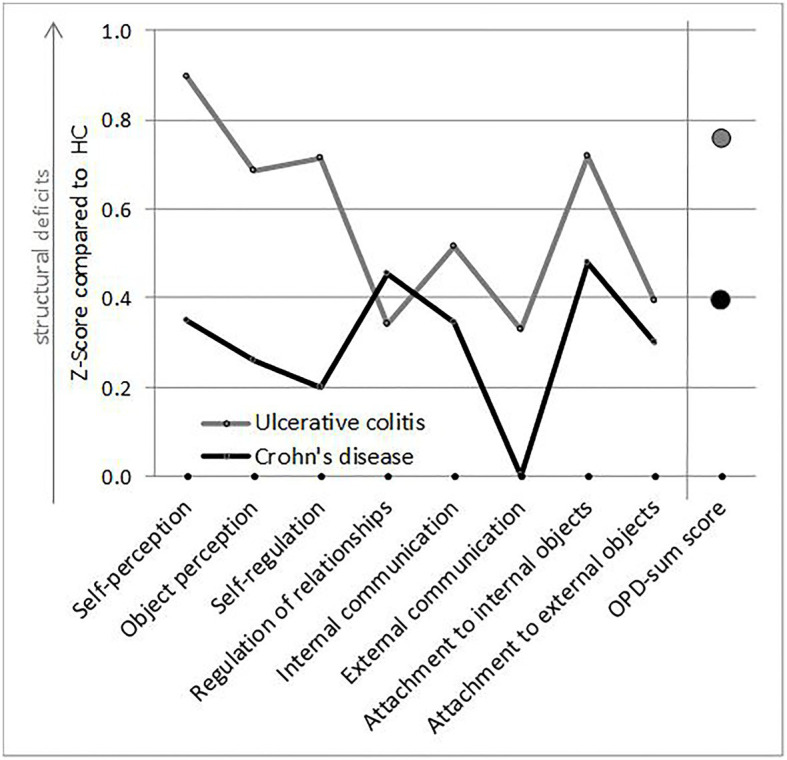
Alterations in psychodynamic structural characteristics in Crohn’s disease (CD) and ulcerative colitis (UC) compared to standardized healthy controls (HCs). The mean values of CD and UC were standardized according to the mean values of HC.

Group comparisons revealed differences in psychological burden (PHQ-9/GAD-7) and psychodynamic structural characteristics (OPD-SQ; see Figure 1 and Supplementary Table S1).

#### Psychological Burden

Depressive and anxiety-related complaints were higher in CD/UC-patients than in HC. Patients with CD/UC reported higher levels of depressive complaints than HC (*d* = 1.0/1.3), and patients with UC reported higher levels of anxiety-related complaints than HC (*d* = 1.1). Small to medium effect sizes were found between CD and HC in anxiety scores (*d* = 0.50) and in group comparison CD/UC (*d* = 0.37).

Based on the established cutoffs, two patients with CD and one patient with UC fulfilled the criteria for a major depressive syndrome. Two patients with CD and three patients with UC fulfilled the criteria for generalized anxiety disorder.

#### Personality Functioning

Patients with CD/UC reported more deficits/alternations in psychodynamic structural characteristics than HC in the total OPD-SQ-score and several primary dimensions of psychodynamic structural characteristics (see also Figure 1). Differences in the overall sum scores were of small to medium effect sizes (sum score *d* = 0.56 for CD/HC; *d* = 0.67 for UC/HC; *d* = 0.20 for UC/CD).

Exploratory analyses of the different psychodynamic structural characteristics revealed different patterns in CD and UC compared to HC.

Patients with CD as well as patients with UC showed differences in self-perception (*d* = 0.59 and *d* = 0.82, respectively). Patients with UC showed differences in self-regulation (*d* = 0.62), internal communication (*d* = 0.52), and attachment to internal objects (*d* = 0.64) with medium effect sizes compared to HC. Other primary dimensions of OPD structure (comparing UC/HC) differed with small effect sizes (*d* = 0.49/0.36/0.28/0.40).

Comparison of CD with HC showed a similar trend, but to a lesser extent. In addition to self-perception, attachment to internal objects differed to HC with a medium effect size (*d* = 0.50), while other effects were small to nonexistent.

Only small differences were found comparing mentalization and attachment (anxiety/avoidance) in CD, UC, and HC (*d* = 0.15 and *d* = 0.11/0.18, respectively). For further data and data analysis, see Supplementary Table S1.

### Correlation Between Personality Functioning and Psychological Burden

Besides the differences *per se* in psychodynamic structural characteristics, depression, and anxiety, the differences correlated with each other. Regarding CD, the OPD-SQ-sum score correlated with the PHQ-9 score [_Sp_*r*: 0.601 (*p* < 0.001)] and with the GAD-7 score [_Sp_*r*: 0.778 (*p* < 0.001)]. For UC, the OPD-SQ-sum score correlated with the PHQ-9 score [_Sp_*r*: 0.523 (*p* = 0.003)] and the GAD-7 score [_Sp_*r*: 0.543 (*p* = 0.002)] for UC. The HC also showed the effect, with a correlation between the OPD-SQ-sum score with the PHQ-9 score with _Sp_*r*: 0.538 (*p* = 0.002) and the GAD-7 score with _Sp_*r*: 0.634 (*p* < 0.001).

## Discussion

The aim of this exploratory study was to describe the psychological burden and personality functioning in patients with CD, UC, and HC. We explored the patterns of various dimensions of psychological distress and personality functioning in comparison with normal “healthy values” in order to have first explorative indications of where the possible deficits might be, and thus provide first ideas for future therapeutic interventions. However, any cause–effect relations cannot be drawn based on the data given in this study. Our results show that the psychological burden and psychodynamic structural characteristics differed between CD/UC and HC with medium to large effect sizes and were correlated with each other. Only little differences were found regarding mentalization and attachment, and comparing CD with UC.

### Psychological Burden: Depressive and Anxiety-Related Complaints

In line with previous research (Gracie et al., 2018), patients with CD/UC reported more depressive complaints than HC, and patients with UC also indicated more anxiety than HC. Using the established severity cutoffs, patients with CD/UC are still in a mild range of anxiety/depressive symptoms and only few meet the criteria of a manifest depressive or anxiety-related syndrome. This is worth mentioning, because patients with CD/UC were in an acute phase of their disease (measured with MIBDI). A previous study showed that IBD patients with active symptoms showed similar psychological burden compared to patients with functional GI complaints, while IBD patients in remission showed a similar psychological burden than HC (Berens et al., 2019). In a previous longitudinal study, it has been shown that CD/UC activity was associated with higher anxiety complaints, and vice versa, i.e., higher anxiety complaints were associated with later flares in IBD (Gracie et al., 2018). This indicates a bidirectional interaction of the (neuro)inflammatory processes and the gut–brain axis. For anxiety and depression, the correlation with psychodynamic structural characteristics – shown in our study – is also interesting. Due to the cross-sectional design, however, we cannot say whether the psychodynamic structural characteristics influenced anxiety/depression or, conversely, whether existing anxiety/depressive symptoms influenced the psychodynamic structural characteristics. Most of the literature sees personality function as a more stable factor, favoring the first explanation (Ehrenthal et al., 2012).

### Personality Functioning – Psychodynamic Structural Characteristics

In our study, patients with CD/UC were characterized by a higher alternation in psychodynamic structural characteristics than the matched HC sample. Since psychodynamic structural characteristics are important for adapting appropriate-to-difficult challenges in life, it is understandable that a deficit in psychodynamic structural characteristics results in a higher psychological burden when experiencing stressful life events (Zimmermann et al., 2012). A higher burden and therefore a higher perceived psychosocial stress can result in an activation of the enteric nervous system, which may lead to a subsequent inflammatory relapse and symptom exacerbation in IBD (Levenstein et al., 2000; Singh et al., 2009). One might speculate that this biopsychosocial link is more relevant for patients with UC than for those with CD, as UC patients are characterized by more structural alternations than CD patients compared to HC. Further research should be undertaken to investigate the influence of psychodynamic structural characteristics on the psychological burden in UC in more detail.

In a further explorative step, we also investigated whether specific dimensions of psychodynamic structural characteristics can be identified that are restricted to CD/UC. We found that compared to HC, patients with CD showed differences in self-perception and attachment to internal objects and that patients with UC showed differences in self-perception, self-regulation, internal communication, and attachment to internal objects – all primary dimensions of the internal world/self. Although these results should certainly be treated with caution due to the exploratory nature of the analyses, they represent an interesting finding, because the structural deficits/alternations seem to be mainly in the area of the self itself and less in the communication/connection toward other people. Dimensions of the internal world/self include items like suffering from inner tension as well as strong and changing feelings. There may also be a connection with flares of UC when the patients indicate that they cannot feel their body properly or do not treat themselves particularly well – a behavior, which could lead to adherence difficulties. Noteworthy, it is interesting that differences in psychodynamic psychological characteristics were more pronounced in UC than in CD. We could not have described these effects, if the groups had been mixed, so we agree with the latest research that the two IBDs should be considered as separate identities to avoid dilution effects (Maunder and Levenstein, 2008).

### Personality Functioning – Mentalization and Attachment

We found only little differences between CD/UC and HC regarding mentalization and attachment.

Since the ability to mentalize has been suggested as a resilience factor for numerous psychological disorders and since various therapeutic approaches to improve the mentalization characteristics exist, the indication of mentalization deficits would have been of direct clinical relevance. Even though our data do not allow final conclusions due to the small sample size and the exploratory character, our results indicate that there are no or only a little mentalization deficits in IBD, when compared to individually matched HCs. At first glance, this seems contradictory to our previous study that reported IBD patients with active symptoms to have higher mentalizing deficits than HC (*d* = 0.4; Berens et al., 2019). Due to the small sample in this study, it would not have been possible to detect small differences. It is noteworthy that in this study, the effect size of patients with UC compared to HC regarding mentalizing deficits was *d* = 0.39 and therefore comparable to the previous study. Thus, it is possible that there are small differences regarding mentalizing in patients with active IBD compared to HC at least in the subgroup of UC patients.

Attachment has already been investigated in several studies. For example, a study found a higher level of anxious and avoidant attachment in IBD patients compared to HC (Caplan et al., 2014), and other studies found a difference in general insecure attachment style regarding UC/CD and HC (Agostini et al., 2010, 2016). Attachment style is generally seen as a stable trait, but chronic diseases like IBD could shift the attachment style, maybe through mentalization deficits triggered by chronic disease, toward insecure attachment (Agostini et al., 2019; Colonnello and Agostini, 2020). These findings seem to be in contrast to our study and might be explained by differences in the way of patient recruitment. While our study includes also patients from secondary care, cited studies focused only on tertiary-care patients (Agostini et al., 2010, 2016, 2019; Caplan et al., 2014). Patients in tertiary care may be more challenged by the difficulties of their disease than patients in secondary care. Furthermore, due to the small sample size, it was not possible to detect small differences. As a trend, patients with CD in our sample seem to be more challenged by attachment-related anxiety (*d* = 0.3) and attachment-related avoidance (*d* = 0.3) than HC. Effect sizes were smaller for UC. Chronic stress, like in IBD, is believed to affect patient’s mentalizing abilities and to determine a shift toward attachment insecurity in patients (Agostini et al., 2019). This possible relationship between mentalizing and attachment was explained in detail in the manuscript of Colonnello and Agostini (2020). Since our patient sample showed only small deficits in mentalization, the shift from secure to insecure attachment could be lower. In general, chronic diseases can be a threatening experience and activate the patients’ attachment systems, leading them in constantly asking for help or relying only on their own strength (Colonnello and Agostini, 2020).

### Strengths and Limitations

The strength of our study is a very precise, individual matching before group comparisons were made. Although this approach significantly reduced sample size, it was the most appropriate way to ensure sufficient comparability between the groups and to control for potential confounding factors. The matching leads to a more realistic picture of differences between CD, UC, and HC without distortion, and the groups remained large enough to detect relevant group differences. Additionally, a realistic picture of patients with IBD could be shown by recruiting from secondary and tertiary care. Still, the duration of symptoms in CD and UC differed, which is probably due to the different onset and peak ages of CD and UC. The limitation of our study is the small sample size with 62 IBD patients (31 per group) due to the strict inclusion/exclusion criteria and the matching. Nevertheless, this was important to eliminate sources of error (such as a worse structure due to differences in disease activity, gender, or age). In general, however, the matching process limits the comparability of the included IBD patients to the general IBD population. Furthermore, the small number of subjects limits the possibility of statistical analysis of mediation factors. In addition, no biological data of the patients (such as C reactive protein or calprotectin) were collected. Thus, it is possible that some of the examined patients have only symptoms without any inflammation activity (IBS-like symptoms) instead of an active IBD. The presence of these IBS-like symptoms is associated with a higher rate of anxiety and depression (Jonefjall et al., 2016; Perera et al., 2019) and therefore may limit our results. When interpreting the results, it must also be considered that the questionnaires for psychodynamic structural characteristics and mentalization used here are relatively new and have not yet been validated in patients with physical diseases. Furthermore, questionnaire data are always subjective and represent only part of the reality, and patients with difficulties in affective processing may not always be aware of their difficulties (Waller and Scheidt, 2006). Therefore, further studies should additionally work with objective criteria, such as performance tests (e.g., the eyes test for mentalization; Vellante et al., 2013; Agostini et al., 2019). However, our data show a high correlation between psychodynamic structural characteristics and anxiety/depression, which at least indicates a clinical significance. It will be the task of further studies to specifically investigate the validity of these instruments in patients with somatic comorbidities. Patients with IBD show an impaired emotion recognition over time, reflecting possible alexithymic traits (Martino et al., 2020). Since there might be patients with alexithymic traits in our sample as well, the values for depression and anxiety might have been underestimated. On the other hand, people with any somatic or psychiatric disease (or intake of antidepressants) were excluded from the “HC” sample, while these exclusion criteria were not used for patients with IBD in order to ensure a representative IBD patient sample. Therefore, there may be an underestimation of the psychological burdens in the control group. Another limitation is that due to the cross-sectional approach and due to normality violations, we could not explore how psychodynamic structural characteristics interact with psychological burden. Further investigations in this direction would require a prospective longitudinal study. But nevertheless, we can provide initial data indicating that a link exists between these two, even if further research is necessary. Furthermore, due to the exploratory nature of this study, we have neither formulated *a priori* hypotheses nor corrected for multiple testing. The statistical analyses presented here are therefore purely descriptive and should not be interpreted as “proving” or statistically conclusive. Future studies are needed to replicate the observations made here in a targeted manner. A larger sample is necessary to ensure that the differences between UC/CD and HC are indeed significant and are not random findings. Nevertheless, it is promising to examine the psychodynamic structural characteristics of the patients in more detail.

### Implications for Further Research

Detailed matching and differentiating between CD and UC seem to make sense for better group comparisons and to avoid dilution effects (Maunder and Levenstein, 2008). For future research, it may be useful to identify CD/UC subgroups with a lower structure in order to investigate whether they are more frequently affected by a severe course of the disease. For the OPD-SQ, there is no general cutoff described; alternatively, the median of the sample could be used (Bugaj et al., 2016). A longitudinal study could address personality functioning, psychological burden, and disease activity in order to determine the impact of one variable on the others. In the future, this could be a more targeted psychological support for IBD patients at risk for a more complicated course of their disease.

### Conclusion

To our knowledge, this is the first report of differences in personality functioning in CD/UC compared to HC. Our data indicate that patients with IBD have a higher level of psychological burden and are more limited in their psychodynamic structural characteristics, while only little differences in mentalization and attachment were found. It seems reasonable that these limitations can also influence the perceived stress and the therapy adherence and, in addition, may possibly influence the disease activity in IBD. Furthermore, our results may suggest a better approach in the psychotherapeutic treatment of IBD.

## Data Availabiltity Statement

Data is available on reasonable request. Requests to access the datasets should be directed to JT, (jonas.tesarz@med.uni-heidelberg.de).

## Ethics Statement

The studies involving human participants were reviewed and approved by the Ethics Committee of the University of Heidelberg and registered by the German Clinical Trials Register (DRKS; DRKS00011685). The patients/participants provided their written informed consent to participate in this study.

## Author Contributions

FE, SB, and JT conceived and designed the study and drafted the manuscript. SB and RS obtained the funding. SB, AG, and JT collected the data. FE and SB statistically analyzed the data. All authors critically revised the manuscript, interpreted the data, and provided important intellectual content. All authors contributed to the article and approved the submitted version.

### Conflict of Interest

The authors declare that the research was conducted in the absence of any commercial or financial relationships that could be construed as a potential conflict of interest.

## Abbreviations

IBD: Inflammatory bowel diseases
CD: Crohn’s disease
UC: Ulcerative colitis
GI: Gastrointestinal
HCs: Healthy controls
IBS: Irritable bowel syndrome
MIBDI: Manitoba inflammatory bowel disease index
Psy-BaDo: Psychosomatic basis documentation questionnaire
PHQ: Patient health questionnaire
GAD: Generalized anxiety disorder questionnaire
OPD: Operationalized psychodynamic diagnosis
OPD-SQ: Operationalized psychodynamic diagnosis—structure questionnaire
MZQ: Mentalization questionnaire
ECR-RD: Experiences in close relationships scale
ISCED: International standard classification of education
SD: Standard deviation
IQR: Interquartile range
_Sp_*r*: Spearman’s rho

## References

[ref1] Agostini A. Ballotta D. Righi S. Moretti M. Bertani A. Scarcelli A.. (2017). Stress and brain functional changes in patients with Crohn’s disease: a functional magnetic resonance imaging study. Neurogastroenterol. Motil. 29, 1–10. 10.1111/nmo.13108, PMID: 28560758

[ref2] Agostini A. Benuzzi F. Filippini N. Bertani A. Scarcelli A. Farinelli V.. (2013a). New insights into the brain involvement in patients with Crohn’s disease: a voxel-based morphometry study. Neurogastroenterol. Motil. 25, 147–e182. 10.1111/nmo.12017, PMID: 22998431

[ref3] Agostini A. Filippini N. Benuzzi F. Bertani A. Scarcelli A. Leoni C.. (2013b). Functional magnetic resonance imaging study reveals differences in the habituation to psychological stress in patients with Crohn’s disease versus healthy controls. J. Behav. Med. 36, 477–487. 10.1007/s10865-012-9441-1, PMID: 22752251

[ref4] Agostini A. Filippini N. Cevolani D. Agati R. Leoni C. Tambasco R.. (2011). Brain functional changes in patients with ulcerative colitis: a functional magnetic resonance imaging study on emotional processing. Inflamm. Bowel Dis. 17, 1769–1777. 10.1002/ibd.21549, PMID: 21744432

[ref5] Agostini A. Rizzello F. Ravegnani G. Gionchetti P. Tambasco R. Straforini G.. (2010). Adult attachment and early parental experiences in patients with Crohn’s disease. Psychosomatics 51, 208–215. 10.1176/appi.psy.51.3.208, PMID: 20484718

[ref6] Agostini A. Scaioli E. Belluzzi A. Campieri M. (2019). Attachment and mentalizing abilities in patients with inflammatory bowel disease. Gastroenterol. Res. Pract. 2019:7847123. 10.1155/2019/7847123, PMID: 31885546 PMC6915150

[ref7] Agostini A. Spuri Fornarini G. Ercolani M. Campieri M. (2016). Attachment and perceived stress in patients with ulcerative colitis, a case-control study. J. Psychiatr. Ment. Health Nurs. 23, 561–567. 10.1111/jpm.12331, PMID: 27624586

[ref8] Berens S. Schaefert R. Baumeister D. Gauss A. Eich W. Tesarz J. (2019). Does symptom activity explain psychological differences in patients with irritable bowel syndrome and inflammatory bowel disease? Results from a multi-center cross-sectional study. J. Psychosom. Res. 126:109836. 10.1016/j.jpsychores.2019.109836, PMID: 31627144

[ref9] Bernstein C. N. (2017). The brain-gut axis and stress in inflammatory bowel disease. Gastroenterol. Clin. N. Am. 46, 839–846. 10.1016/j.gtc.2017.08.006, PMID: 29173525

[ref10] Bugaj T. J. Müksch C. Ehrenthal J. C. Köhl-Hackert N. Schauenburg H. Huber J.. (2016). Stress in medical students: a cross-sectional study on the relevance of attachment style and structural integration. Psychother. Psychosom. Med. Psychol. 66, 88–92. 10.1055/s-0035-1569285, PMID: 26859112

[ref11] Burisch J. Jess T. Martinato M. Lakatos P. L. (2013). The burden of inflammatory bowel disease in Europe. J. Crohns Colitis 7, 322–337. 10.1016/j.crohns.2013.01.010, PMID: 23395397

[ref12] Caplan R. A. Maunder R. G. Stempak J. M. Silverberg M. S. Hart T. L. (2014). Attachment, childhood abuse, and IBD-related quality of life and disease activity outcomes. Inflamm. Bowel Dis. 20, 909–915. 10.1097/MIB.0000000000000015, PMID: 24651585

[ref13] Charlson M. E. Pompei P. Ales K. L. Mackenzie C. R. (1987). A new method of classifying prognostic comorbidity in longitudinal studies: development and validation. J. Chronic Dis. 40, 373–383. 10.1016/0021-9681(87)90171-8, PMID: 3558716

[ref14] Clara I. Lix L. M. Walker J. R. Graff L. A. Miller N. Rogala L.. (2009). The Manitoba IBD index: evidence for a new and simple indicator of IBD activity. Am. J. Gastroenterol. 104, 1754–1763. 10.1038/ajg.2009.197, PMID: 19455122

[ref15] Cohen J. (1998). Statistical Power Analysis for the Behavioral Sciences. Hillsdale, NJ: Lawrence Erlbaum Associates.

[ref16] Colonnello V. Agostini A. (2020). Disease course, stress, attachment, and mentalization in patients with inflammatory bowel disease. Med. Hypotheses 140:109665. 10.1016/j.mehy.2020.109665, PMID: 32155541

[ref17] de Souza H. S. P. (2017). Etiopathogenesis of inflammatory bowel disease: today and tomorrow. Curr. Opin. Gastroenterol. 33, 222–229. 10.1097/MOG.0000000000000364, PMID: 28402995

[ref18] Ehrenthal J. C. Dinger U. Horsch L. Komo-Lang M. Klinkerfuß M. Grande T.. (2012). The OPD structure questionnaire (OPD-SQ): first results on reliability and validity. Psychother. Psychosom. Med. Psychol. 62, 25–32. 10.1055/s-0031-1295481, PMID: 22271173

[ref19] Fan Y. Bao C. Wei Y. Wu J. Zhao Y. Zeng X.. (2020). Altered functional connectivity of the amygdala in Crohn’s disease. Brain Imaging Behav. 14, 2097–2106. 10.1007/s11682-019-00159-8, PMID: 31628591

[ref20] Fischer-Kern M. Tmej A. (2019). Mentalization and depression: theoretical concepts, treatment approaches and empirical studies—an overview. Z. Psychosom. Med. Psychother. 65, 162–177. 10.13109/zptm.2019.65.2.162, PMID: 31154932

[ref21] Fonagy P. Target M. (2006). The mentalization-focused approach to self pathology. J. Personal. Disord. 20, 544–576. 10.1521/pedi.2006.20.6.544, PMID: 17192138

[ref22] Gracie D. J. Guthrie E. A. Hamlin P. J. Ford A. C. (2018). Bi-directionality of brain-gut interactions in patients with inflammatory bowel disease. Gastroenterology 154, 1635–1646.e3. 10.1053/j.gastro.2018.01.027, PMID: 29366841

[ref23] Hausberg M. C. Schulz H. Piegler T. Happach C. G. Klopper M. Brutt A. L.. (2012). Is a self-rated instrument appropriate to assess mentalization in patients with mental disorders? Development and first validation of the mentalization questionnaire (MZQ). Psychother. Res. 22, 699–709. 10.1080/10503307.2012.709325, PMID: 22867004

[ref24] Hayden M. C. Mullauer P. K. Gaugeler R. Senft B. Andreas S. (2018). Improvements in mentalization predict improvements in interpersonal distress in patients with mental disorders. J. Clin. Psychol. 74, 2276–2286. 10.1002/jclp.22673, PMID: 29998458 PMC6282818

[ref25] Heuft G. Senf W. Bell K. Cording C. Geyer M. Janssen P. L.. (1998). Psy-BaDo kernmodul einer basisdokumentation in der fachpsychotherapie. Psychotherapeut 43, 48–52. 10.1007/s002780050099

[ref26] Hou J. Mohanty R. Nair V. A. Dodd K. Beniwal-Patel P. Saha S.. (2019). Alterations in resting-state functional connectivity in patients with Crohn’s disease in remission. Sci. Rep. 9:7412. 10.1038/s41598-019-43878-0, PMID: 31092855 PMC6520362

[ref27] Ibm Corp (2016). “IBM SPSS Statistics for Windows”. 24.0 ed. (Armonk, NY: IBM Corp).

[ref28] Jonefjall B. Ohman L. Simren M. Strid H. (2016). IBS-like symptoms in patients with ulcerative colitis in deep remission are associated with increased levels of serum cytokines and poor psychological well-being. Inflamm. Bowel Dis. 22, 2630–2640. 10.1097/MIB.0000000000000921, PMID: 27636379

[ref29] Kielholz P. (1977). Mental illness and stress. Schweiz. Arch. Neurol. Neurochir. Psychiatr. 121, 9–19. PMID: 23580

[ref30] Kragel P. A. Kano M. Van Oudenhove L. Ly H. G. Dupont P. Rubio A.. (2018). Generalizable representations of pain, cognitive control, and negative emotion in medial frontal cortex. Nat. Neurosci. 21, 283–289. 10.1038/s41593-017-0051-7, PMID: 29292378 PMC5801068

[ref31] Kroenke K. Spitzer R. L. Williams J. B. (2001). The PHQ-9: validity of a brief depression severity measure. J. Gen. Intern. Med. 16, 606–613. 10.1046/j.1525-1497.2001.016009606.x, PMID: 11556941 PMC1495268

[ref32] Ladwig B. (2016). Wie lässt sich das Bindungsverhalten von Patientinnen mit Anorexia Nervosa charakterisieren und welche Rolle spielen dabei traumatische Kindheitserlebnisse? Albert-Ludwigs-University.

[ref33] Lafontaine M.-F. Brassard A. Lussier Y. Valois P. Shaver P. R. Johnson S. M. (2016). Selecting the best items for a short-form of the experiences in close relationships questionnaire. Eur. J. Psychol. Assess. 32, 140–154. 10.1027/1015-5759/a000243

[ref34] Leiner D. J. (2014). “SoSci survey [Computer software]”.

[ref35] Lenhard W. Lenhard A. (2016). Berechnung von Effektstärken [Online]. Dettelbach: Psychometrica. Available at: https://www.psychometrica.de/effektstaerke.html (Accessed February, 2020).

[ref36] Levenstein S. Prantera C. Varvo V. Scribano M. L. Andreoli A. Luzi C.. (2000). Stress and exacerbation in ulcerative colitis: a prospective study of patients enrolled in remission. Am. J. Gastroenterol. 95, 1213–1220. 10.1111/j.1572-0241.2000.02012.x, PMID: 10811330

[ref37] Löwe B. Spitzer R. Zipfel S. Herzog W. (2002). PHQ-D–Gesundheitsfragebogen für Patienten, Manual–Komplettversion und Kurzform–Autorisierte deutsche Version des “Prime MD Patient Health Questionnaire (PHQ),” [Patient Health Questionnaire, Manual—Full and short Version—authorized German Version of the Prime MD Patient Health Questionnaire (PHQ)]. Heidelberg: Pfizer.

[ref38] Luyten P. Fonagy P. (2018). The stress–reward–mentalizing model of depression: an integrative developmental cascade approach to child and adolescent depressive disorder based on the research domain criteria (RDoC) approach. Clin. Psychol. Rev. 64, 87–98. 10.1016/j.cpr.2017.09.008, PMID: 29107398

[ref39] Martino G. Caputo A. Schwarz P. Bellone F. Fries W. Quattropani M. C.. (2020). Alexithymia and inflammatory bowel disease: a systematic review. Front. Psychol. 11:1763. 10.3389/fpsyg.2020.01763, PMID: 32973596 PMC7466427

[ref40] Maunder R. G. Levenstein S. (2008). The role of stress in the development and clinical course of inflammatory bowel disease: epidemiological evidence. Curr. Mol. Med. 8, 247–252. 10.2174/156652408784533832, PMID: 18537632

[ref41] Mikulincer M. Shaver P. R. (2003). “The attachment behavioral system in adulthood: activation, psychodynamics, and interpersonal processes,” in Advances in experimental social psychology. ed. Zanna M. P. (USA: Elsevier Science), 53–152.

[ref42] Muskin P. R. (2014). DSM-5 Self-Exam Questions: Test Questions for the Diagnostic Criteria. Washington, DC; London, England: American Psychiatric Pub.

[ref43] Perera L. P. Radigan M. Guilday C. Banerjee I. Eastwood D. Babygirija R.. (2019). Presence of irritable bowel syndrome symptoms in quiescent inflammatory bowel disease is associated with high rate of anxiety and depression. Dig. Dis. Sci. 64, 1923–1928. 10.1007/s10620-019-05488-8, PMID: 30725303

[ref44] Persoons P. Luyckx K. Fischler B. (2001). Psychiatric diagnoses in gastroenterology: validation of a self-report instrument (PRIME-MD patient health questionnaire), epidemiology and recognition. Gastroenterology 120:A114. 10.1016/S0016-5085(01)80560-6

[ref45] Petruo V. A. Krauss E. Kleist A. Hardt J. Hake K. Peirano J.. (2019). Perceived distress, personality characteristics, coping strategies and psychosocial impairments in a national German multicenter cohort of patients with Crohn’s disease and ulcerative colitis. Z. Gastroenterol. 57, 473–483. 10.1055/a-0838-6371, PMID: 30965377

[ref46] Sajadinejad M. S. Asgari K. Molavi H. Kalantari M. Adibi P. (2012). Psychological issues in inflammatory bowel disease: an overview. Gastroenterol. Res. Pract. 2012:106502. 10.1155/2012/106502, PMID: 22778720 PMC3388477

[ref47] Schojai M. (2017). Zusammenhang zwischen Bindungserfahrung und späterem Beziehungsverhalten bei Patienten mit Borderline-Persönlichkeitsstörung. Ruhr University Bochum.

[ref48] Singh S. Graff L. A. Bernstein C. N. (2009). Do NSAIDs, antibiotics, infections, or stress trigger flares in IBD? Am. J. Gastroenterol. 104, 1298–1313. 10.1038/ajg.2009.15, PMID: 19337242

[ref49] Skrobisz K. Piotrowicz G. Naumczyk P. Sabisz A. Markiet K. Rydzewska G.. (2020). Imaging of morphological background in selected functional and inflammatory gastrointestinal diseases in fMRI. Front. Psychol. 11:461. 10.3389/fpsyt.2020.00461, PMID: 32508692 PMC7251141

[ref50] Spitzer R. L. Kroenke K. Williams J. B. Loewe B. (2006). A brief measure for assessing generalized anxiety disorder: the GAD-7. Arch. Intern. Med. 166, 1092–1097. 10.1001/archinte.166.10.1092, PMID: 16717171

[ref51] Szabo S. Yoshida M. Filakovszky J. Juhasz G. (2017). “Stress” is 80 years old: From Hans Selye original paper in 1936 to recent advances in GI ulceration. Curr. Pharm. Des. 23, 4029–4041. 10.2174/1381612823666170622110046, PMID: 28641541

[ref52] Tasca G. A. Brugnera A. Baldwin D. Carlucci S. Compare A. Balfour L.. (2018). Reliability and validity of the experiences in close relationships scale-12: attachment dimensions in a clinical sample with eating disorders. Int. J. Eat. Disord. 51, 18–27. 10.1002/eat.22807, PMID: 29215748

[ref53] Thomann A. K. Schmitgen M. M. Kmuche D. Ebert M. P. Thomann P. A. Szabo K.. (2020). Exploring joint patterns of brain structure and function in inflammatory bowel diseases using multimodal data fusion. Neurogastroenterol. Motil. 33:e14078. 10.1111/nmo.14078, PMID: 33368950

[ref54] Turkiewicz J. Bhatt R. R. Wang H. Vora P. Krause B. Sauk J. S.. (2021). Altered brain structural connectivity in patients with longstanding gut inflammation is correlated with psychological symptoms and disease duration. Neuroimage Clin. 30:102613. 10.1016/j.nicl.2021.102613, PMID: 33823388 PMC8050027

[ref55] Unesco Institute for Statistics (2012). “International Standard Classification of Education: ISCED 2011.” (Montreal, Canada).

[ref56] Vellante M. Baron-Cohen S. Melis M. Marrone M. Petretto D. R. Masala C.. (2013). The “Reading the mind in the eyes” test: systematic review of psychometric properties and a validation study in Italy. Cogn. Neuropsychiatry 18, 326–354. 10.1080/13546805.2012.721728, PMID: 23106125 PMC6345369

[ref57] Vogt B. A. (2013). Inflammatory bowel disease: perspectives from cingulate cortex in the first brain. Neurogastroenterol. Motil. 25, 93–98. 10.1111/nmo.12067, PMID: 23336589

[ref58] Waller E. Scheidt C. E. (2006). Somatoform disorders as disorders of affect regulation: a development perspective. Int. Rev. Psychiatry 18, 13–24. 10.1080/09540260500466774, PMID: 16451876

[ref59] Yeung A. W. K. (2021). Structural and functional changes in the brain of patients with Crohn’s disease: an activation likelihood estimation meta-analysis. Brain Imaging Behav. 15, 807–818. 10.1007/s11682-020-00291-w, PMID: 32333318

[ref60] Zikou A. K. Kosmidou M. Astrakas L. G. Tzarouchi L. C. Tsianos E. Argyropoulou M. I. (2014). Brain involvement in patients with inflammatory bowel disease: a voxel-based morphometry and diffusion tensor imaging study. Eur. Radiol. 24, 2499–2506. 10.1007/s00330-014-3242-6, PMID: 25001084

[ref61] Zimmermann J. Ehrenthal J. C. Cierpka M. Schauenburg H. Doering S. Benecke C. (2012). Assessing the level of structural integration using operationalized psychodynamic diagnosis (OPD): implications for DSM–5. J. Pers. Assess. 94, 522–532. 10.1080/00223891.2012.700664, PMID: 22808938

