# Supplementary material for: Higher Levels of Psychological Burden and Alterations in Personality Functioning in Crohn’s Disease and Ulcerative Colitis
Source: Front Psychol. 2021 Jun 24;12:671493. doi: 10.3389/fpsyg.2021.671493 (PMC8264053; doi:10.3389/fpsyg.2021.671493)
Supplement: Supplementary file 1 [file Table_1.pdf]

Supplementary material

Table S1: Psychodynamic structural characteristics and psychological burden

	Crohn's disease n=31		ulcerative colitis n=31		healthy controls n=31		comparison CD/UC/HC	comparison CD/HC	comparison UC/HC	comparison CD/UC
**Personality functioning**										
Psychodynamic structural characteristics (OPD-SQ; 0-4)[a]	31	0.94 (0.81)	30	1.15 (0.99)	31	0.72 (0.63)	$\chi^2(2)=7.464$<br>p=0.024<br>d=0.512	U(1)=331.000<br>p=0.035<br>d=0.555	U(1)=293.000<br>p=0.013<br>d=0.670	U(1)=412.000<br>p=0.445<br>d=0.197
Self-perception[a]	31	0.49 (0.83)	30	0.88 (1.08)	31	0.25 (0.42)	$\chi^2(2)=9.939$<br>p=0.007<br>d=0.626	U(1)=323.500<br>p=0.027<br>d=0.585	U(1)=260.500<br>p=0.003<br>d=0.816	U(1)=389.000<br>p=0.272<br>d=0.284
Object-perception[a]	31	0.82 (1.10)	30	1.12 (1.03)	31	0.65 (0.65)	$\chi^2(2)=3.826$<br>p=0.148<br>d=0.289	U(1)=375.000<br>p=0.137<br>d=0.384	U(1)=337.000<br>p=0.065<br>d=0.487	U(1)=445.500<br>p=0.778<br>d=0.072
Self-regulation[a]	31	0.83 (1.08)	30	1.19 (1.19)	31	0.69 (0.92)	$\chi^2(2)= 5.212$<br>p=0.074<br>d=0.387	U(1)=409.500<br>p=0.317<br>d=0.256	U(1)=304.500<br>p=0.020<br>d=0.621	U(1)=382.000<br>p=0.231<br>d=0.310
Regulation of relationships[a]	31	0.92 (0.67)	30	0.83 (1.29)	31	0.58 (1.08)	$\chi^2(2)=2.946$<br>p=0.229<br>d=0.207	U(1)=369.500<br>p=0.118<br>d=0.405	U(1)=368.500<br>p=0.163<br>d=0.362	U(1)=463.000<br>p=0.977<br>d=0.007
Internal communication[a]	31	1.00 (0.64)	30	1.09 (0.91)	31	0.82 (0.73)	$\chi^2(2)=4.620$<br>p=0.099<br>d=0.348	U(1)=365.000<br>p=0.103<br>d=0.422	U(1)=328.000<br>p=0.048<br>d=0.523	U(1)=425.500<br>p=0.568<br>d=0.146
External communication[a]	31	1.29 (0.71)	30	1.46 (0.73)	31	1.29 (0.79)	$\chi^2(2)=2.114$<br>p=0.348<br>d=0.072	U(1)=452.500<br>p=0.693<br>d=0.100	U(1)=390.000<br>p=0.279<br>d=0.280	U(1)=371.000<br>p=0.175<br>d=0.353
Attachment to internal objects[a]	31	1.00 (1.00)	30	1.19 (1.00)	31	0.63 (1.25)	$\chi^2(2)=6.390$<br>p=0.041<br>d=0.456	U(1)=346.000<br>p=0.058<br>d=0.496	U(1)=301.000<br>p=0.018<br>d=0.636	U(1)=426.500<br>p=0.578<br>d=0.143
Attachment to external objects[a]	31	1.63 (0.88)	30	1.50 (1.28)	31	1.25 (1.13)	$\chi^2(2)=3.593$<br>p=0.166<br>d=0.270	U(1)=358.000<br>p=0.084<br>d=0.449	U(1)=358.500<br>p=0.124<br>d=0.403	U(1)=463.500<br>p=0.983<br>d=0.006

Mentalization (MZQ; 0-4)[a]	31	2.07 (1.00)	29	2.13 (1.00)	31	1.73 (1.07)	$\chi^2(2)=2.475$ p=0.290 d=0.147	U(1)=458.000 p=0.751 d=0.081	U(1)=349.500 p=0.139 d=0.389	U(1)=368.500 p=0.230 d=0.313
Attachment (ECR-RD 12; 0-7)										
Attachment related anxiety (ECR-RD 12; 0-7)[a]	29	1.38 (1.46)	30	1.58 (1.67)	31	1.67 (1.67)	$\chi^2(2)=1.743$ p=0.418 d=0.109	U(1)=361.000 p=0.185 d=0.343	U(1)=421.000 p=0.522 d=0.163	U(1)=392.500 p=0.509 d=0.168
Attachment related avoidance (ECR-RD 12; 0-7)[a]	29	2.00 (1.83)	30	1.50 (1.38)	31	1.50 (1.50)	$\chi^2(2)=1.350$ p=0.509 d=0.174	U(1)=377.000 p=0.279 d=0.280	U(1)=458.000 p=0.919 d=0.026	U(1)=375.000 p=0.359 d=0.239
Psychological burden										
Depression (PHQ-9; 0-27)[a]	30	6.5 (6.00)	31	6.00 (5.00)	31	2.00 (3.00)	$\chi^2(2)=21.144$ p<0.001 d=1.047	U(1)=220.000 p<0.001 d=1.015	U(1)=174.000 p<0.001 d=1.31	U(1)=442.500 p=0.744 d=0.083
Anxiety (GAD-7; 0-21)[a]	31	4.00 (10.00)	31	6.00 (6.00)	31	2.00 (4.00)	$\chi^2(2)=14.08$ p=0.001 d=0.787	U(1)=345.000 p=0.053 d=0.499	U(1)=204.500 p<0.001 d=1.135	U(1)=378.500 p=0.149 d=0.371

OPD-SQ: operationalized psychodynamic diagnosis- Structure Questionnaire; MZQ: Mentalization Questionnaire; ECR_RD: Experiences in Close Relationships Scale; PHQ-9: patient health questionnaire; GAD-7; Generalized Anxiety Disorder questionnaire; Due to normality violations, p-values were calculated with Kruskal-Wallis-Tests (three groups), respectively Mann-Whitney-U-tests (two groups).; d=Cohen's d; [a]median and interquartile range (IQR). Note: Due to the exploratory approach of this study, we didn't correct for multiple testing and p-values therefore should be interpreted with caution. A total of six tests (for psychodynamic structural characteristics, mentalization, attachment (2), depression and anxiety) were carried out, eight post-hoc-test per group (UC/CD/HC) for the different primary dimensions of psychodynamic structural characteristics.
